# CRISPR Applications in Alzheimer’s Disease: From High-Throughput Genetic Screening to Precision Editing and CNS Delivery

**DOI:** 10.3390/ijms27083371

**Published:** 2026-04-09

**Authors:** You Li, Shixin Ma, Teng Fei

**Affiliations:** 1 Key Laboratory of Bioresource Research and Development of Liaoning Province, College of Life and Health Sciences, Northeastern University, Shenyang 110819, China; nanjiliyou@163.com (Y.L.); mashixin0902@163.com (S.M.); 2 Foshan Graduate School of Innovation, Northeastern University, Foshan 528311, China; 3 National Frontiers Science Center for Industrial Intelligence and Systems Optimization, Northeastern University, Shenyang 110819, China; 4 Key Laboratory of Data Analytics and Optimization for Smart Industry (Northeastern University), Ministry of Education, Shenyang 110819, China

**Keywords:** Alzheimer’s disease, CRISPR, disease modeling, high-throughput screening, gene editing

## Abstract

Alzheimer’s disease is a devastating progressive neurodegenerative disorder characterized by extracellular amyloid-beta plaques and intracellular tau tangles. Despite recent advancements in amyloid-beta-targeting immunotherapies, achieving safe and definitive disease control remains a profound clinical challenge. The CRISPR/Cas9 system has emerged as a powerful technology for precision neurogenetics, offering significant potential to address the fundamental questions behind Alzheimer’s disease. This comprehensive review delineates the trajectory of CRISPR applications in Alzheimer’s disease research and therapeutics. First, we explore the integration of CRISPR in engineering high-fidelity in vitro models, such as isogenic induced pluripotent stem cells and three-dimensional cerebral organoids, alongside advanced in vivo mammalian models. Second, we examine how these platforms facilitate unbiased high-throughput genetic screening to uncover molecular underpinnings regulating tau, lipid metabolism, and neuroinflammation. Third, we critically evaluate precision editing strategies targeting core risk genes (*APP*, *MAPT*, *APOE*, and *TREM2*), explicitly highlighting the severe physiopathological trade-offs between therapeutic efficacy and loss-of-function toxicity. Finally, we address the ultimate translational bottlenecks impeding clinical application. By dissecting the packaging limits of adeno-associated viral vectors and the physical barricade of the blood–brain barrier, we underscore the necessity of transitioning toward next-generation base editors and non-viral lipid nanoparticles to realize safe and efficacious in vivo clinical gene therapies against Alzheimer’s disease.

## 1. Introduction

Alzheimer’s disease (AD) is a devastating progressive neurodegenerative disorder and the primary cause of dementia in the elderly population worldwide. This condition imposes an increasing socioeconomic burden on global healthcare systems [1]. The complex pathogenesis of AD is defined by two signature proteinopathies: extracellular amyloid-beta (Aβ) plaque deposition and the intracellular accumulation of neurofibrillary tangles composed of hyperphosphorylated tau proteins [2,3]. Conventional pharmacological interventions have largely failed to improve the clinical trajectory of the disease despite decades of extensive research [4]. Current therapies, including cholinesterase inhibitors and glutamate regulators, offer only transient symptomatic relief without halting neuronal death [1]. Although Aβ-targeting monoclonal antibodies such as aducanumab and lecanemab recently received accelerated approval, their clinical benefits remain modest and are often coupled with serious adverse events [5]. These immunotherapies are frequently associated with amyloid-related imaging abnormalities (ARIA), which include cerebral edema and microhemorrhages, thus limiting their therapeutic windows [5]. Furthermore, the physical and biochemical properties of the blood–brain barrier (BBB) obstruct the efficient delivery of systemic small molecules and biologics to the central nervous system (CNS) [6]. These limitations necessitate the development of innovative, disease-modifying modalities like precision gene editing to target the fundamental genetic abnormalities driving AD [7].

The CRISPR/Cas9 (clustered regularly interspaced short palindromic repeats/CRISPR-associated protein 9) system has emerged as a powerful and versatile genome engineering technology [8]. Derived from an adaptive immune mechanism in bacteria and archaea, the CRISPR/Cas9 platform utilizes a programmable single guide RNA (sgRNA) to direct the Cas9 endonuclease to specific DNA sequences [9,10]. After recognizing a protospacer adjacent motif (PAM), Cas9 induces targeted double-strand breaks (DSBs), which are subsequently repaired via non-homologous end joining (NHEJ) or homology-directed repair (HDR) [9,11]. CRISPR/Cas9 provides superior operational simplicity and multiplexing capabilities compared to earlier nucleases such as ZFNs and TALENs [6]. Next-generation CRISPR tools, including base editors and prime editors, further allow for precise single-nucleotide conversions without generating toxic DSBs [12]. Engineered Cas variants, such as catalytically inactive “dead” Cas9 (dCas9) and Cas9 nickases (nCas9), enable targeted epigenetic modulation and reversible gene regulation [13]. Additionally, novel temporal control mechanisms using light-activated sgRNAs provide refined spatiotemporal regulation. These advancements reduce off-target risks and establish a safer profile for clinical interventions [14].

**Figure 1:**
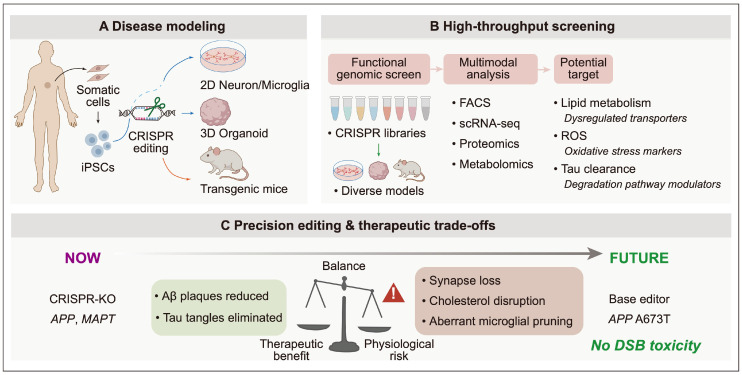
The comprehensive CRISPR/Cas9 pipeline in Alzheimer’s disease research: from disease modeling and high-throughput screening to precision genome editing. (**A**) Disease modeling: Somatic cells from AD patients are reprogrammed into induced pluripotent stem cells (iPSCs) and edited utilizing CRISPR technology to generate isogenic 2D neural cultures and 3D cerebral organoids. Additionally, CRISPR is employed directly to generate transgenic mouse models. (**B**) High-throughput screening: Pooled or arrayed CRISPR libraries are applied to these models, leveraging fluorescence-activated cell sorting (FACS) and single-cell RNA sequencing (scRNA-seq) to uncover novel therapeutic targets governing lipid metabolism, reactive oxygen species (ROS) production, and tau clearance. (**C**) Precision editing and therapeutic trade-offs: The current paradigm utilizing conventional CRISPR-KO to target core genes (e.g., *APP*, *MAPT*) is depicted as a physiological balance, weighing therapeutic benefits (reductions in Aβ plaques and tau tangles) against severe physiological risks (synapse loss and aberrant microglial pruning). To overcome these limitations, the pipeline evolves towards next-generation base editing (e.g., the protective *APP* A673T insertion), which offers a superior, non-destructive therapeutic strategy by circumventing double-strand break (DSB) toxicity.

The application of CRISPR/Cas technologies in AD research is a rapidly evolving field. While recent comprehensive reviews have excellently detailed its foundational mechanisms, general pathology, and broad target landscape [15], our manuscript is specifically designed to address a complementary niche: the profound translational gap between in vitro therapeutic efficacy and in vivo physiopathological realities. To provide a specialized and clinically pragmatic perspective, this comprehensive review delineates the trajectory of CRISPR/Cas9 applications in AD from fundamental discovery to clinical translation. First, we discuss the role of CRISPR in engineering sophisticated in vitro platforms, such as patient-derived induced pluripotent stem cells (iPSCs), and faithful in vivo mammalian models [16]. Second, we examine how these models facilitate unbiased CRISPR screening for the high-throughput discovery and validation of novel pathogenic drivers or targets. Third, we evaluate next-generation precision editing strategies for core pathological drivers including *APP*, apolipoprotein E (*APOE*), and *TREM2* [17,18]. We explicitly evaluate the “double-edged sword” of these targets, emphasizing the severe physiopathological trade-offs and biological costs of complete ablation that must be addressed during clinical translation to prevent unintended neuroimmune consequences [19,20]. Finally, we address the ultimate translational bottleneck of CNS delivery. We conclude that penetrating the BBB and overcoming the 4.7 kb packaging limits of adeno-associated viral (AAV) vectors are the essential prerequisites for establishing efficacious clinical gene therapies for AD [21]. The comprehensive CRISPR/Cas9 research pipeline, encompassing disease modeling, target screening, and precision therapeutic editing, is illustrated in Figure 1.

## 2. Application of CRISPR in AD Modeling

### 2.1. Genetic Targets for AD Modeling: Familial vs. Sporadic Drivers

The genetic landscape of AD serves as the fundamental roadmap for constructing physiologically relevant CRISPR models. AD is clinically and genetically categorized into two major forms: early-onset familial AD (fAD) and late-onset sporadic AD (sAD) [22]. fAD is primarily driven by autosomal dominant mutations in three key genes: the amyloid precursor protein (*APP*), presenilin 1 (*PSEN1*), and presenilin 2 (*PSEN2*) [23]. Mutations in *PSEN1* and *PSEN2*, which encode the catalytic subunits of the γ-secretase complex, lead to the overproduction of pathogenic Aβ42 species [24]. In contrast, sAD is highly polygenic, with the *APOE4* allele and variants in microglial genes, notably *TREM2*, serving as major genetic risk factors [25]. Understanding these distinct genetic targets is a prerequisite for selecting appropriate CRISPR modeling strategies.

### 2.2. Overview of AD Models

Accurately capturing the complex and multifactorial pathogenesis of AD, including the aforementioned amyloid and tau proteinopathies, necessitates robust and physiologically relevant disease models [26]. The multifactorial etiology of AD requires robust disease models, which are currently categorized into in vitro platforms, such as patient-derived iPSCs and three-dimensional (3D) organoids, and in vivo mammalian models, predominantly consisting of genetically modified mice [27,28]. Historically, in vivo transgenic models relied on the artificial overexpression of fAD mutations to simulate proteinopathies, although this approach often introduced non-physiological artifacts. The advent of CRISPR technologies has revolutionized the construction of these models by facilitating precise genomic modifications. CRISPR enables the creation of in vivo knock-in (KI) and knock-out (KO) animal models that accurately replicate human genetic variations at endogenous levels, thereby bypassing the limitations associated with traditional overexpression paradigms [28].

### 2.3. iPSCs and In Vitro Cell Models

The integration of CRISPR into patient-derived iPSC research has transformed in vitro disease modeling. Investigating late-onset sporadic AD risk variants is frequently complicated by inter-individual genetic heterogeneity [26]. CRISPR/Cas9 mitigates this challenge through the creation of isogenic control cell lines, which are genetically identical to the parental cells except at the specific targeted risk locus [29]. This approach grants researchers the distinct advantage of eliminating genetic background noise.

For instance, researchers have utilized CRISPR/Cas9 to convert the disease-neutral *APOE3* allele into the *APOE4* risk variant in healthy donor iPSCs, or conversely to correct *APOE4* to *APOE3* in patient-derived cells [29]. These isogenic models revealed that the *APOE4* variant inherently drives elevated Aβ generation in neurons, impairs Aβ clearance in astrocytes, and disrupts lipid metabolism. Crucially, the conversion of *APOE4* to *APOE3* was sufficient to alleviate most AD-related phenotypes in these patient-derived models [30]. Furthermore, CRISPR technology has been applied to address gene dosage effects in familial AD, such as amyloid precursor protein (*APP*) duplication. By utilizing highly specific paired Cas9 nickases to progressively inactivate distinct alleles, researchers engineered isogenic clones with varying functional *APP* dosages, demonstrating that correcting gene dosage significantly reduces Aβ secretion and tau hyperphosphorylation [18]. Stable KO models for other risk genes, including microtubule-associated protein tau (*MAPT*), *SORL1*, and triggering receptor expressed on myeloid cells 2 (*TREM2*), have further uncovered essential roles for these genes in mediating amyloid toxicity and immune dysfunction [31,32].

### 2.4. 3D Brain Organoid Models

Although 2D iPSC cultures offer cellular insights, they lack the spatial complexity and cytoarchitecture of the human brain. To better simulate the AD brain microenvironment, CRISPR-edited iPSCs have been induced for differentiation into 3D cerebral organoids, which facilitate the study of complex cell–cell interactions within a physiological matrix. Organoids generated from *APOE4* isogenic iPSCs exhibit elevated Aβ and tau pathology after extended culture, successfully recapitulating age-dependent AD pathologies more effectively than 2D systems [29]. Additionally, CRISPR/Cas9 has been used to delete the *ABCA7* gene in healthy iPSCs to create 3D cortical organoids [33]. Functional analyses of these engineered organoids provided novel evidence that *ABCA7* deficiency alters mitochondrial lipid metabolism and exacerbates neuronal dysregulation within a neural network [33].

### 2.5. In Vivo Animal Models

In the realm of in vivo modeling, CRISPR has facilitated a transition from conventional transgenic mice to novel KI and KO models that mirror authentic human disease genetics [34]. Compared to traditional homologous recombination, CRISPR technologies significantly enhance the efficiency of animal model construction by streamlining the targeting process and reducing the timelines required for germline transmission.

The rapid construction of targeted AD mice using CRISPR is now well-documented. For example, the humanized *TREM2* R47H variant has been introduced into murine models to study microglial responses [35]. One model generated via CRISPR-mediated insertion of the human *TREM2* R47H sequence on an APP/PS1 background revealed that this mutation attenuates microglial responses to amyloid plaques. Beyond germline editing, CRISPR is also employed to engineer human cells prior to their transplantation into murine brains to create advanced chimeric models [34]. In a recent breakthrough, CRISPR/Cas9 was used to edit human iPSC-derived microglia to express the Aβ-degrading enzyme neprilysin under the control of the plaque-responsive *CD9* promoter [34]. When xenografted into mice, these CRISPR-engineered microglia achieved pathology-responsive delivery of therapeutic proteins, successfully lowering insoluble Aβ levels and preserving neuronal density in vivo [34]. To provide a clear comparison of the various platforms used in AD research, the specific CRISPR applications, key advantages, and inherent limitations of 2D iPSCs, 3D organoids, and in vivo rodent models are systematically summarized in Table 1.

## 3. Applications of High-Throughput CRISPR Screening in AD Target Discovery

### 3.1. Overview of CRISPR Screening Technologies

The advent of genome-wide and targeted CRISPR screening technologies has provided a revolutionary framework for discovering novel therapeutic targets in AD [6]. These high-throughput platforms, encompassing CRISPR/Cas9 knockout (CRISPR-KO), CRISPR interference (CRISPRi), and CRISPR activation (CRISPRa) libraries, enable the systematic and multiplexed functional interrogation of thousands of genes simultaneously [36]. Unlike traditional candidate-based approaches that rely on hypothesis-driven assumptions, pooled and arrayed CRISPR screens facilitate unbiased, genome-scale explorations of genetic risk factors and complex regulatory networks [6]. This technological paradigm shift fundamentally transitions AD research from studying isolated candidate genes to comprehensively mapping the molecular pathways associated with neurodegeneration. The distinct mechanisms and primary applications of different high-throughput CRISPR screening modalities, including knockout, interference, and activation libraries for AD target discovery, are categorized and compared in Table 2.

### 3.2. CRISPR Screening in 2D Cell Models

Given that AD pathogenesis is highly dependent on cell type-specific responses, applying CRISPR screens in 2D cellular models has yielded significant discoveries. In human primary astrocytes, a large-scale CRISPRi screen targeting 979 distal candidate enhancers successfully mapped functional regulatory interactions controlling AD-associated genes, such as the astrocyte-enriched *LGALS3* gene [36]. In parallel, targeted CRISPRi screens conducted in human iPSC-derived microglia utilized reactive oxygen species (ROS) production in response to the viral mimic poly(I:C) to identify inflammatory modulators [37]. This screen revealed that silencing the AD risk genes *MS4A6A* and *EED* amplified ROS production and pro-inflammatory states, whereas knocking down *INPP5D* or *RABEP1* blunted the ROS response [37]. Furthermore, an arrayed CRISPR/Cas9 knockout screen in both *APOE3-*expressing and *APOE*-KO iPSC-derived microglia uncovered that the mTORC1 signaling pathway acts as a crucial negative regulator of lipid droplet accumulation [38]. Beyond glial cells, a genome-wide CRISPR-KO screen performed in human *Ngn2*-induced cortical excitatory neurons successfully identified potent regulators of endogenous tau protein levels, specifically highlighting the ubiquitin-proteasome system and mTOR pathway components like *TSC1* and *TSC2* [39].

### 3.3. CRISPR Screening in 3D Organoid Models

While 2D systems provide robust high-throughput capabilities, 3D brain organoid models represent the next frontier for CRISPR screening by mimicking complex neural networks and spatial intercellular interactions. Recent advancements have successfully integrated CRISPR/Cas9 systems with optogenetic controls in human iPSC-derived midbrain organoids to induce pathological protein aggregation, creating reliable 3D platforms for neurodegenerative drug screening [40]. Additionally, patient-derived AD-specific cerebral organoids carrying mutations generated via CRISPR/Cas9 offer highly physiological environments to study disease mechanisms in a multicellular context. Building on this foundation, emerging research since 2024 has actively deployed targeted CRISPR interference (CRISPRi) and knockout screens within these 3D models [41]. For instance, screens focusing on tau propagation have identified novel kinase dependencies within a 3D neural network, while the co-culture of iPSC-derived microglia with organoids has enabled specialized CRISPR-KO screens targeting neuroinflammation and microglial phagocytosis in response to amyloid plaques [42].

Although large-scale pooled screens in 3D AD organoids possess immense potential to unveil how genetic perturbations influence progressive AD pathologies within a spatially organized tissue architecture, they are currently limited by profound technical complexities. Implementing these pooled screens faces substantial bottlenecks, including high batch-to-batch structural heterogeneity, inherently low lentiviral library delivery efficiencies into deep tissue layers, and the general immaturity of organoid-derived neurons. Moreover, the typical lack of functional vascularization and comprehensive peripheral neuro-immune interactions in standard organoid models hinders the full physiological translation of screening hits. Overcoming these delivery and structural barriers will be critical for utilizing 3D organoids in genome-wide AD target discovery.

### 3.4. In Vivo CRISPR Screening in Animal Models

In vivo CRISPR screening represents a critical methodological advancement wherein viral expression vectors coding for Cas9 and sgRNA libraries are delivered directly into the living mouse brain via stereotaxic injection. AAV vectors are predominantly used for this central nervous system delivery due to their stability, heterogeneous tissue infection capabilities, and low immunogenicity. This in vivo approach is paramount because it allows researchers to interrogate genetic targets under the authentic physiological conditions of an aging microenvironment, an intact blood–brain barrier, and a complete physiological immune system. For example, candidate genes initially identified in cellular screens, such as *TSC1*, have been successfully validated in vivo, proving that conditionally knocking out these targets directly in the live mammalian brain actively increases tau protein accumulation [39]. Ultimately, deploying multiplexed CRISPR libraries directly into living animal brains enables the discovery of disease-modifying physiological targets that simply cannot be captured or replicated in artificial in vitro cell culture.

## 4. Precision Editing: Therapeutic Strategies and Biological Hurdles

### 4.1. Targeting the Amyloid and Tau Pathways: Efficacy vs. Physiological Function

Recent advances in human iPSC modeling have provided a robust platform for demonstrating the efficacy of CRISPR/Cas9 in correcting genetic drivers of AD [43]. Precision genome editing has successfully targeted autosomal dominant mutations in the *APP*, presenilin 1 (*PSEN1*), and presenilin 2 (*PSEN2*) genes. For instance, CRISPR/Cas9-mediated disruption of the *APP* Swedish mutation (*APP*swe) in patient-derived fibroblasts resulted in a robust 60% reduction in secreted Aβ40 [44]. Similarly, targeted correction of the *PSEN1* M146L allele and the *PSEN2* N141I mutation effectively normalized the pathogenic Aβ42/40 ratio and rescued critical electrophysiological deficits in human cholinergic neurons [45,46]. Furthermore, targeting the *MAPT* gene has demonstrated that CRISPR-mediated chronic tau depletion in iPSC-derived cortical neurons significantly mitigates Aβ-driven toxicity and neuronal hyperactivity [47].

Nevertheless, extrapolating these highly controlled in vitro successes to in vivo therapeutic applications exposes significant physiological hurdles. Genes such as *APP* and *MAPT* are not merely pathological substrates. They also govern indispensable functions in normal neurobiology. Total ablation of *APP* in human neuronal models triggers cholesterol-associated developmental defects, impairs synaptogenesis, and severely alters synaptic vesicle dynamics, highlighting its essential physiological role in neuronal maintenance [48]. Parallel consequences are observed with tau ablation, where complete *MAPT* KO or depletion yields neurons with decreased activity and compromised neurite outgrowth [47]. Beyond the danger of target loss-of-function, the mechanics of traditional CRISPR/Cas9 editing pose severe safety liabilities in living tissue. Relying on DSBs to permanently alter the genome of post-mitotic neurons in vivo introduces the risk of off-target cleavage, unintended chromosomal rearrangements, and the potential to trigger an intense host immune response against the Cas9 protein [19]. Consequently, employing permanent gene ablation to clear amyloid or tau in a living neural network may inadvertently substitute proteinopathic toxicity with severe physiopathological and genomic failure. To mitigate the severe synaptic consequences of complete *APP* or *MAPT* ablation, translational strategies are shifting toward highly precise, non-destructive modalities. These include the use of neuron-specific promoters to restrict editing exclusively to pathogenic cell populations, or the deployment of Cas9 nickases to perform allele dosage titration, effectively reducing Aβ burden while preserving basal physiological functions. Most notably, base editing strategies designed to introduce the protective *APP* A673T mutation offer a promising paradigm to inhibit amyloidogenic processing without inducing loss-of-function toxicity [49].

### 4.2. Modulating Lipid Metabolism and Neuroinflammation: The APOE and TREM2 Dilemmas

Beyond amyloid and tau, precision editing has provided elegant in vitro paradigms for investigating the neuroimmune and lipid axes of AD. The *APOE4* allele remains the most significant genetic risk factor for sporadic AD, driving early neuronal maturation, altering astrocyte lipid metabolism, and impairing glial amyloid clearance. Utilizing gene editing to convert the high-risk *APOE4* allele into the neutral *APOE3* variant has proven highly efficacious in 3D cerebral organoids, successfully attenuating Aβ accumulation and tau pathology while reversing most disease-associated features [29,50]. Similarly, CRISPR/Cas9-mediated modeling of the *TREM2* gene, a critical regulator of microglial activation, has elucidated key neuroinflammatory mechanisms. Introducing the *TREM2* Y38C variant into murine models successfully demonstrated its role in impairing synaptic plasticity and myelination [51].

Despite these in vitro achievements, translating lipid and neuroinflammatory modulators in vivo introduces a complex biological dilemma. Simplistic knockout strategies have risks of disrupting the brain’s fundamental homeostatic infrastructure. While some early-stage models suggest that knocking out *APOE* might prevent cellular senescence in vitro [52], the permanent absence of *APOE* in vivo could severely disrupt cholesterol transport essential for myelin repair and synaptic plasticity. Furthermore, manipulating microglial checkpoints like *TREM2* acts as a double-edged sword. While some paradigms aim to dampen neuroinflammation, complete *TREM2* deletion paradoxically exacerbates tau accumulation and accelerates brain atrophy in the presence of amyloid pathology [53]. Additional studies in *Trem2*-null mice have shown that the loss of this receptor leads to delayed and abnormal microglial activation following inflammatory challenges [54]. Permanently shifting the immunological state of microglia in vivo carries the risk of either inducing uncontrolled neuroinflammation or triggering aberrant synaptic pruning, highlighting the danger of utilizing irreversible knockout strategies for dynamic or context-dependent regulators. Given the double-edged nature of neuroimmune modulation, irreversible knockout strategies for genes like *TREM2* are clinically perilous. Instead, researchers are developing dynamic regulatory approaches, such as microglia-specific conditional editing utilizing the *CX3CR1* promoter. Furthermore, CRISPR interference and activation (CRISPRi/CRISPRa) systems are being explored to reversibly titrate *TREM2* expression across different disease stages [55]. Future clinical applications may also leverage pathology-responsive promoters to restrict CRISPR expression strictly to the proximity of amyloid plaques, thereby fine-tuning microglial activation without disrupting global CNS immune homeostasis.

### 4.3. The Next Frontier: Base Editing and Epigenetic Modulation

To circumvent the physiological hazards of complete gene ablation and the severe genotoxicity of DSBs, the field is rapidly pivoting toward next-generation precision technologies. Base editing enables precise single-base transitions without severing the DNA double helix. This non-destructive approach has been successfully employed to insert the protective *APP* A673T mutation into human cell lines (HEK293T and SH-SY5Y), which significantly reduces the accumulation of toxic Aβ40 and Aβ42 species while avoiding the synaptic consequences of total *APP* knockout [49]. Alternatively, highly specific paired Cas9 nickases have been strategically applied to titrate *APP* gene dosage by inactivating specific alleles. This approach restores physiological neuronal function and reduces apoptosis without strictly eliminating the entire gene pool [26]. On the other hand, CRISPR interference (CRISPRi), which leverages catalytically dead Cas9 (dCas9), has emerged as a robust tool for temporal epigenetic modulation. A recently optimized drug-inducible CRISPR ON/OFF system utilizing dCas9 has demonstrated the capability to dynamically regulate microglial genes associated with AD [55]. This allows researchers to modulate disease-associated variants and neuroinflammation without inducing DSBs or causing irreversible genomic rearrangements.

A comprehensive comparison of the therapeutic benefits and corresponding physiological risks associated with diverse CRISPR strategies across core AD genetic targets is summarized in Table 3. While base editing and dCas9-mediated epigenetic regulation theoretically bypass the genotoxicity and permanent loss-of-function dilemmas associated with traditional CRISPR/Cas9, they introduce a formidable translational hurdle for in vivo therapy. The molecular architectures of base editors and dCas9 fusion proteins are exceptionally massive, significantly exceeding the ~4.7 kilobase packaging capacity of standard AAV vectors [56]. Due to the large size of the commonly used *Streptococcus pyogenes* Cas9 variant, delivering these bulky molecular machineries across the BBB and into the complex tissues of the CNS requires advanced strategies, such as complex viral vectors or non-viral nanocomplexes [19]. Without a safe and highly efficient in vivo delivery mechanism that targets the appropriate cell populations while bypassing reticuloendothelial clearance, the vast therapeutic promise of non-destructive precision editing remains largely confined to the in vitro setting. This critical bottleneck must be resolved before bringing these therapies to the clinic.

## 5. CNS Delivery: Overcoming Physical and Immunological Barriers

The translation of advanced gene-editing therapeutics from bench to bedside holds significant potential for the treatment of intractable neurodegenerative and neurogenetic disorders [61]. However, the CNS represents one of the most challenging pharmacological targets in human physiology [62]. Successful in vivo genome intervention strictly requires delivery platforms that are capable of navigating complex biological terrains without eliciting severe host toxicity [63]. In this chapter, we delineate the profound translational bottlenecks governing CNS gene delivery, dissect the precise physical and immunological barriers, and evaluate both established viral vectors and frontier non-viral paradigms [64].

### 5.1. The Blood–Brain Barrier and CRISPR Immunogenicity

The most formidable physical obstacle to neuro-targeted gene therapy is the BBB, a highly specialized and selective semipermeable border comprising brain microvascular endothelial cells, pericytes, and astrocyte endfeet [61]. Tethered together by intricate tight junction complexes, the BBB severely restricts paracellular transport, creating a physical bottleneck that naturally excludes the systemic diffusion of macromolecular gene-editing tools, such as CRISPR/Cas ribonucleoproteins (RNPs) and therapeutic nucleic acids [65]. Consequently, this natural barricade inherently prevents systemically administered therapies from reaching therapeutic bioavailability in the brain parenchyma [66].

Beyond the physical blockade of the BBB, a critical immunological barrier arises from the bacterial origin of the most widely utilized genome-editing systems. Enzymes such as *Streptococcus pyogenes* Cas9 (SpCas9) and *Staphylococcus aureus* Cas9 (SaCas9) are fundamentally foreign antigens to the human immune system [67]. Upon in vivo expression or delivery, these bacteria-derived Cas proteins can provoke severe adaptive immune responses [62]. In a large proportion of the human population, pre-existing humoral and cellular immunity can rapidly neutralize the editing machinery or trigger the targeted destruction of successfully transduced neurons. This acquired immune rejection not only dampens the long-term therapeutic efficacy of the intervention but also carries the risk of precipitating lethal neuroinflammation, severely undermining the safety profile of CRISPR-based therapies in clinical settings [68].

### 5.2. AAV Vectors and the “Packaging Limit” Crisis

Recombinant adeno-associated viruses (rAAVs) currently dominate the landscape of CNS gene therapy due to their favorable safety profile, episomal persistence, and robust ability to transduce post-mitotic neurons [61]. Over the past decade, significant successes have been achieved through the use of neurotropic serotypes, notably AAV9, and next-generation engineered variants such as AAV-PHP.B and AAV-PHP.eB [69]. These capsids demonstrate an exceptional receptor-mediated capacity to cross the intact BBB following intravenous administration and achieve widespread transduction of neuronal and glial populations across the CNS [70].

However, a fundamental translational bottleneck of AAV-mediated precision genome editing lies in the packaging limit. The biological architecture of the AAV capsid imposes a strict nucleic acid packaging capacity of approximately 4.7 kilobases (kb) [71]. This physical restriction is in conflict with the massive size of next-generation genomic tools. Base editors, prime editors and epigenome editors, which fuse catalytically impaired Cas proteins to large effector domains, possess coding sequences that drastically exceed the 4.7 kb threshold, making single-vector packaging a biological impossibility.

To circumvent this payload restriction, researchers have engineered dual-AAV (split-AAV) systems [72]. In this approach, the massive editing complex is fragmented into two separate AAV vectors, utilizing split-intein technology to facilitate post-translational protein trans-splicing and reconstitution of the full enzyme within the target cell [73]. Unfortunately, this strategy is plagued by severe inefficiencies in vivo. For a successful genomic edit to occur, a single neuron must be simultaneously co-transduced by both distinct AAV vectors at high multiplicity, followed by the seamless transcription, translation, and spatial reconstitution of the protein fragments [72]. In the vast and complex architecture of the living brain, this multi-step prerequisite drastically diminishes overall recombination efficiency, leading to suboptimal editing efficiencies that often fall below the therapeutic threshold required for phenotypic rescue.

While adeno-associated viruses (AAVs) remain the most widely used vectors for in vivo CNS delivery, the diverse requirements of AD gene therapy necessitate the evaluation of alternative viral platforms, notably adenoviral and lentiviral vectors.

Adenoviral vectors offer a significant advantage regarding payload capacity (up to 36 kb), easily accommodating massive CRISPR/Cas9 systems and multiple guide RNAs within a single virion. Previous studies have demonstrated the potential of directing adenoviruses across the BBB via the melanotransferrin (P97) transcytosis pathway in in vitro models [74], highlighting their utility for systemic CNS targeting. However, their clinical translation for AD is severely hindered by high immunogenicity, relatively low transduction efficiency in mature CNS neurons compared to glia, and the transient nature of episomal expression in dividing cell populations, which often provokes robust inflammatory responses.

Lentiviral vectors, conversely, represent a robust option for establishing stable long-term transgene expression in non-dividing cells, such as post-mitotic neurons [75]. Their ability to integrate large genetic payloads (up to 8–10 kb) makes them highly advantageous for delivering complex base editors or multiplexed CRISPR arrays. They are particularly invaluable for ex vivo engineering of patient-derived cells or generating stable in vitro disease models. Nonetheless, for in vivo therapeutic applications, lentiviral vectors carry inherent risks of insertional mutagenesis and oncogene activation. Ensuring the long-term safety of integrating vectors in the human CNS remains a paramount clinical barrier that must be rigorously addressed before widespread therapeutic adoption.

### 5.3. Non-Viral Nanocarriers and Cargo Modalities

Beyond the choice of viral or non-viral vehicles, the physical modality of the CRISPR/Cas9 cargo critically determines editing efficiency and safety. Currently, CRISPR components can be delivered in three primary forms: plasmid DNA, mRNA, and pre-assembled ribonucleoproteins (RNPs) [10]. Delivery via plasmid DNA ensures robust Cas9 expression, but this extended duration significantly exacerbates the risk of off-target cleavage. Alternatively, mRNA-based delivery provides a safer and transient expression profile without genomic integration risks [76]. Most recently, the delivery of RNP complexes (Cas9 protein directly bound to sgRNA) has emerged as the gold standard for precision editing. RNPs enable immediate target cleavage followed by rapid proteolytic degradation—a transient “hit-and-run” mechanism that dramatically minimizes off-target effects [77].

Non-viral nanocarriers, most notably lipid nanoparticles (LNPs) and synthetic ribonucleoprotein (RNP) complexes, have emerged as promising and versatile platforms for CNS delivery [78]. LNPs offer a distinct advantage by providing an essentially limitless payload capacity, seamlessly accommodating the delivery of large mRNAs encoding base or prime editor modalities [79]. Furthermore, non-viral vectors facilitate transient “hit-and-run” expression of the editing machinery [64]. By delivering mRNA or RNPs that degrade rapidly after executing their genomic edits, LNPs drastically reduce the temporal window for off-target genomic cleavage and minimize the presentation of bacterial Cas peptides to the host immune system, thereby circumventing the severe immunogenicity associated with persistent viral expression [80].

Despite these advantages, facilitating the penetration of LNPs across the BBB remains a challenge. To break this barrier, contemporary neuroengineering has pivoted toward highly sophisticated targeted delivery strategies [81]. One leading approach relies on receptor-mediated transcytosis (RMT), wherein the surface of the nanoparticle is functionalized with specific BBB shuttle peptides or monoclonal antibodies [82]. For example, customized BBB-crossing peptides, such as PB5-3, have been structurally designed through phage display to specifically bind transcytosis-competent receptors on the luminal surface of brain microvascular endothelial cells [83]. These targeted ligands actively shuttle both viral and non-viral cargoes into the deep brain without compromising the structural integrity of the endothelial tight junctions [84].

**Figure 2:**
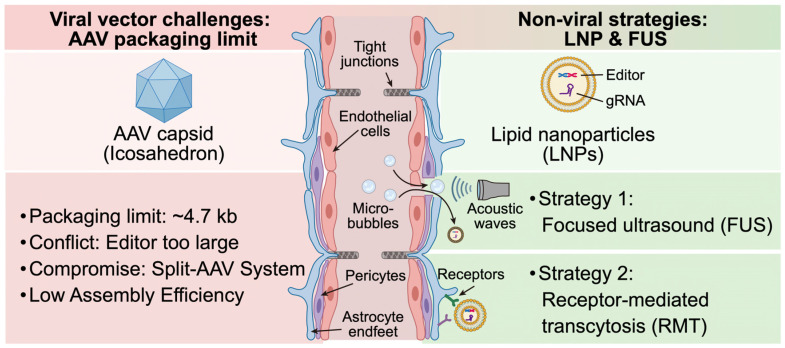
Overcoming translational bottlenecks in CNS delivery: AAV packaging limits and non-viral blood–brain barrier penetration strategies. The central structure depicts the blood–brain barrier (BBB), a formidable physical defense composed of endothelial cells, tight junctions, pericytes, and astrocyte endfeet. The left panel illustrates the inherent limitations of adeno-associated viral (AAV) vectors, specifically the ~4.7 kb packaging limit that physically restricts the delivery of massive base or prime editors, as well as the suboptimal assembly efficiency of split-AAV systems. The right panel demonstrates advanced non-viral strategies utilizing lipid nanoparticles (LNPs) to bypass these cargo constraints. LNPs can traverse the BBB either via transient tight junction permeabilization induced by focused ultrasound (FUS) combined with microbubbles (Strategy 1), or via receptor-mediated transcytosis (RMT) (Strategy 2). These innovative platforms enable the efficient delivery of large genomic editors and guide RNAs into the central nervous system.

Concurrently, physical modulation techniques offer a revolutionary non-invasive approach to bypass the BBB [61]. Focused ultrasound (FUS), combined with the systemic administration of microbubbles, represents a cutting-edge strategy to achieve transient and localized BBB opening [85]. The targeted acoustic waves induce the rapid oscillation and stable cavitation of the microbubbles within the cerebral microvasculature, generating mechanical forces that temporarily dismantle the tight junctions between endothelial cells [86]. This highly controlled and reversible permeabilization allows systemically administered LNPs or viral vectors to extravasate directly into specific and pre-determined regions of the brain parenchyma [87]. By synergizing advanced non-viral nanocarriers with targeted biochemical transcytosis or ultrasound-mediated permeabilization, the field is steadily overcoming the historical barriers of CNS delivery, paving the way for safe and highly efficient neurogenetic interventions [88]. The contrasting physical barriers, viral packaging limits, and advanced non-viral penetration strategies for CNS delivery are summarized in Figure 2.

## 6. Conclusions and Future Perspectives

Over the past decade, the CRISPR/Cas system has evolved from mere molecular scissors to a comprehensive genome editing toolkit. In the AD scenario, this technology has been applied for high-throughput genetic screening, sophisticated modeling of familial and sporadic AD mechanisms, and precision editing for targeted therapeutic interventions. Nevertheless, the clinical translation of these tools is currently constrained by major technical and biological barriers. Technically, the reliance on classical active nucleases that induce permanent DSBs inherently carries profound risks of genomic instability, unpredictable off-target mutagenesis, and severe physiological toxicity [89]. Furthermore, the physical barrier of the BBB severely restricts CNS access of current delivery vehicles. Further limitations of delivery systems include constrained payload capacities and immunogenic profiles for AAVs [19], and the suboptimal CNS biodistribution and transient efficacy for LNPs. Biologically, the multifactorial pathogenesis of AD, coupled with irreversible neuronal loss by the time of clinical diagnosis, presents a profound hurdle that complicates any singular genetic intervention. By critically evaluating these physiopathological trade-offs, synthesizing high-throughput screening advancements, and dissecting specific CNS delivery bottlenecks, this review aims to systematically bridge the translational gap between in vitro efficacy and in vivo realities.

To overcome the genotoxicity of DSBs and bridge the gap to clinical translation, “next-generation” editing tools are being actively explored. Base Editing (BE) and Prime Editing (PE) represent significant future opportunities. For instance, the in vivo conversion of the highly penetrant *APOE4* risk allele to the neutral *APOE3* or protective *APOE2* variant using base editors has shown considerable preclinical utility, offering genetic modification without toxic DNA cleavage. Furthermore, CRISPR interference (CRISPRi) for the targeted epigenetic silencing of dosage-sensitive genes like *APP* or *MAPT* (tau) is rapidly advancing. By modulating gene expression without altering the underlying genomic sequence, CRISPRi circumvents the primary safety hurdles of traditional Cas9 nucleases, positioning it as a viable candidate for near-future clinical evaluation. Crucially, these next-generation strategies are now advancing into higher-order species. Recent developments have demonstrated the successful in vivo application of base editors in non-human primates (NHPs), revealing robust CNS editing efficiencies and long-term tolerability, which provides critical large-animal validation required for clinical progression [90]. Furthermore, while registered clinical trials for CRISPR-based AD therapies are in nascent stages, several pipeline programs utilizing transient LNP delivery for neurodegenerative targets are rapidly advancing toward clinical translation and Investigational New Drug (IND) evaluation.

Looking ahead, continued technological refinements and concerted global efforts are required. Conquering the CNS delivery bottleneck mandates cross-disciplinary collaboration between material science and neuroengineering to develop tropism-directed non-viral nanoparticles and evolved AAV variants that are capable of navigating the BBB. Although clinical trials for CRISPR-based AD therapies have yet to be initiated, the recent success of NTLA-2001 [91], a systemically administered lipid nanoparticle-based in vivo CRISPR therapy for transthyretin (ATTR) amyloidosis, provides valuable clinical proof-of-concept that safely clearing certain proteinopathies via systemic gene editing is technically achievable. With more efficient and safer modalities along with improved delivery vehicles, bridging the gap between technical feasibility and therapeutic efficacy in AD remains profoundly challenging. While CRISPR technology may not offer a singular definitive cure, with improved delivery vehicles, it holds significant potential to accelerate basic research and contribute to future multifaceted therapeutic strategies for Alzheimer’s disease.

## Figures and Tables

**Table 1:** Summary of CRISPR-Engineered Alzheimer’s Disease Models.

Model System	CRISPR Application in AD Research	Key Advantages	Current Limitations
2D iPSC-derived Neurons/Glia	Introducing or correcting fAD mutations (e.g., *PSEN1*, *APP*) to study early cellular phenotypes.	Human genetic background; highly scalable for high-throughput genetic screening.	Lacks complex 3D tissue architecture and comprehensive neuro-immune interactions.
3D Cerebral Organoids	Modeling Aβ plaque deposition and tau tangle formation in a spatial context.	Recapitulates spatial cytoarchitecture and early developmental pathophysiology.	High batch-to-batch variability; typically lacks functional vascularization and mature microglia.
In vivo Rodent Models	Generating humanized knock-in models (e.g., *MAPT*, *APOE4*) or rapid somatic targeted knockouts.	Enables vital behavioral, cognitive, and systemic physiological assessments.	Species-specific biological differences; lengthy generation timelines for germline editing.

**Table 2:** Overview of High-Throughput CRISPR Screening Modalities in AD Target Discovery.

Screening Modality	Mechanism of Action	Primary Application in AD Research	Key Advantages
CRISPR-KO (Knockout)	Induces double-strand breaks (DSBs) resulting in frameshift mutations and complete gene ablation.	Identifying essential genetic dependencies and novel therapeutic targets (e.g., discovering survival factors under Aβ toxicity).	Provides robust, permanent loss-of-function phenotypes; ideal for identifying absolute genetic drivers.
CRISPRi (Interference)	Employs catalytically dead Cas9 (dCas9) fused to transcriptional repressors (e.g., KRAB) to block transcription.	Silencing dosage-sensitive AD genes (e.g., *APP*, *MAPT*) or transiently downregulating immune regulators.	Reversible and precise down-regulation without inducing toxic DNA damage or genomic instability.
CRISPRa (Activation)	Employs dCas9 fused to transcriptional activators (e.g., VPR) to upregulate endogenous gene expression.	Identifying protective factors, neurotrophic drivers, or genes that enhance microglial clearance of Aβ plaques.	Enables physiological gain-of-function studies of large genes that exceed the payload limits of standard viral vectors.

**Table 3:** CRISPR-Mediated Genome Editing in Alzheimer’s Disease: Therapeutic Benefits vs. Physiological Risks.

Target Gene & Variant	CRISPR Strategy	Therapeutic Benefits	Physiopathological Risks & Hurdles	Refs.
*APP* (*APP*swe, *APP* WT)	CRISPR-KO, Base Editing (A673T insertion), Cas9 nickase	Robustly reduces toxic Aβ peptide accumulation (Aβ40/42). Correcting gene dosage decreases Aβ secretion, tau hyperphosphorylation, and apoptotic pathways.	Total *APP* knockout impairs synaptogenesis and severely alters synaptic vesicle dynamics. Causes cholesterol-associated developmental deficits (e.g., reduced neurite growth).	[26,44,48,49]
*MAPT* (tau)	CRISPR-KO (Exon 1 or 4 depletion)	Chronic tau depletion mitigates Aβ-driven toxicity. Protects against neuronal hyperactivity and halts synapse loss.	Complete tau depletion negatively impacts normal basal neuronal activity. Leads to compromised neurite outgrowth and shortened axon initial segments.	[47,57]
*APOE* (*APOE4*)	CRISPR-KI (*APOE4* to *APOE3* conversion), CRISPR-KO	Converting the high-risk *APOE4* allele to *APOE3* reverses AD-like changes, reducing Aβ/tau accumulation. *APOE* KO may prevent cellular senescence in vitro.	Permanent deletion risks destroying the brain’s lipid homeostasis and cholesterol transport infrastructure. Threatens fundamental mechanisms vital for neuronal support, synaptic plasticity, and myelin repair.	[29,50,52]
*TREM2* (*TREM2*-null, *TREM2* Y38C)	CRISPR-KO, CRISPR-KI (Y38C mutation)	Targeted editing modulates neuroinflammation and microglial activation. Wild-type *TREM2* plays a protective role in clearing aggregates.	A physiological double-edged sword: deletion paradoxically worsens tau accumulation and accelerates brain atrophy in the presence of amyloid pathology. Loss of function delays microglial activation and impairs myelination.	[51,53,54]
*PSEN1* (PS1 ΔE9, PS1 L150P)	CRISPR-KI (Mutation correction), Base Editing	Successfully corrects fAD mutations in patient iPSCs. Robustly normalizes the Aβ42/40 ratio and reduces extracellular Aβ aggregates.	As a core catalytic subunit of γ-secretase, complete knockout severely impairs Notch signaling. Risks disrupting vital proteolytic processing of multiple substrates essential for neuronal development.	[58,59,60]
*PSEN2* (PS2 N141I)	CRISPR-KI (N141I correction)	Restores Aβ42/40 balance in fAD cholinergic neurons. Ameliorates electrophysiological deficits and calcium dysregulation in edited cells.	Though less severe than *PSEN1* loss, it still threatens the overall stability of the γ-secretase complex. Potential for off-target effects in homologous regions of *PSEN1* given their structural similarity.	[15,46]

## Data Availability

No new data were created or analyzed in this study. Data sharing is not applicable to this article.

## Abbreviations

The following abbreviations are used in this manuscript:

2D	Two-dimensional
3D	Three-dimensional
AAV	Adeno-associated virus/Adeno-associated viral
AD	Alzheimer’s disease
APOE	Apolipoprotein E
APP	Amyloid precursor protein
ARIA	Amyloid-related imaging abnormalities
ATTR	Transthyretin
Aβ	Amyloid-beta
BBB	Blood–brain barrier
CNS	Central nervous system
CRISPR	Clustered regularly interspaced short palindromic repeats
CRISPRa	CRISPR activation
CRISPRi	CRISPR interference
dCas9	Catalytically inactive “dead” Cas9
DSB	Double-strand break
FACS	Fluorescence-activated cell sorting
fAD	Familial Alzheimer’s disease
FUS	Focused ultrasound
HDR	Homology-directed repair
iPSC	Induced pluripotent stem cell
kb	Kilobase
KI	Knock-in
KO	Knock-out
LNP	Lipid nanoparticle
MAPT	Microtubule-associated protein tau
nCas9	Cas9 nickase
NHEJ	Non-homologous end joining
PAM	Protospacer adjacent motif
PSEN1	Presenilin 1
PSEN2	Presenilin 2
rAAV	Recombinant adeno-associated virus
RMT	Receptor-mediated transcytosis
RNP	Ribonucleoprotein
ROS	Reactive oxygen species
SaCas9	Staphylococcus aureus Cas9
scRNA-seq	Single-cell RNA sequencing
sgRNA	Single guide RNA
SpCas9	Streptococcus pyogenes Cas9
TALENs	Transcription activator-like effector nucleases
TREM2	Triggering receptor expressed on myeloid cells 2
ZFNs	Zinc-finger nucleases

## References

[1] Chacko L., Chaudhary A., Singh B., Dewanjee S., Kandimalla R. (2023). CRISPR-Cas9 in Alzheimer’s disease: Therapeutic trends, modalities, and challenges. Drug Discov. Today.

[2] LaFerla F.M., Oddo S. (2005). Alzheimer’s disease: Abeta, tau and synaptic dysfunction. Trends Mol. Med..

[3] Scheltens P., Blennow K., Breteler M.M., de Strooper B., Frisoni G.B., Salloway S., Van der Flier W.M. (2016). Alzheimer’s disease. Lancet.

[4] Kalra R.S., Dhanjal J.K., Das M., Singh B., Naithani R. (2021). Cell Transdifferentiation and Reprogramming in Disease Modeling: Insights into the Neuronal and Cardiac Disease Models and Current Translational Strategies. Cells.

[5] Cavazzoni P. FDA’s Decision to Approve New Treatment for Alzheimer’s Disease. https://web.archive.org/web/20221206233519/https://www.fda.gov/drugs/news-events-human-drugs/fdas-decision-approve-new-treatment-alzheimers-disease.

[6] Meshram H.K., Gupta S.K., Gupta A., Nagori K., Ajazuddin (2025). Next-generation CRISPR gene editing tools in the precision treatment of Alzheimer’s and Parkinson’s disease. Ageing Res. Rev..

[7] Anzalone A.V., Koblan L.W., Liu D.R. (2020). Genome editing with CRISPR-Cas nucleases, base editors, transposases and prime editors. Nat. Biotechnol..

[8] Mullard A. (2020). Gene-editing pipeline takes off. Nat. Rev. Drug Discov..

[9] Barrangou R., Horvath P. (2017). A decade of discovery: CRISPR functions and applications. Nat. Microbiol..

[10] Liu C., Zhang L., Liu H., Cheng K. (2017). Delivery strategies of the CRISPR-Cas9 gene-editing system for therapeutic applications. J. Control. Release.

[11] Du J., Yin N., Xie T., Zheng Y., Xia N., Shang J., Chen F., Zhang H., Yu J., Liu F. (2018). Quantitative assessment of HR and NHEJ activities via CRISPR/Cas9-induced oligodeoxynucleotide-mediated DSB repair. DNA Repair.

[12] Komor A.C., Kim Y.B., Packer M.S., Zuris J.A., Liu D.R. (2016). Programmable editing of a target base in genomic DNA without double-stranded DNA cleavage. Nature.

[13] Qi L.S., Larson M.H., Gilbert L.A., Doudna J.A., Weissman J.S., Arkin A.P., Lim W.A. (2013). Repurposing CRISPR as an RNA-guided platform for sequence-specific control of gene expression. Cell.

[14] De Plano L.M., Calabrese G., Conoci S., Guglielmino S.P.P., Oddo S., Caccamo A. (2022). Applications of CRISPR-Cas9 in Alzheimer’s Disease and Related Disorders. Int. J. Mol. Sci..

[15] Khan M.S., Qureshi N., Khan R., Son Y.O., Maqbool T. (2025). CRISPR/Cas9-Based therapeutics as a promising strategy for management of Alzheimer’s disease: Progress and prospects. Front. Cell. Neurosci..

[16] Wang Y., Wang Y., Bharti V., Zhou H., Hoi V., Tan H., Wu Z., Nagakannan P., Eftekharpour E., Wang J.F. (2019). Upregulation of Thioredoxin-Interacting Protein in Brain of Amyloid-beta Protein Precursor/Presenilin 1 Transgenic Mice and Amyloid-beta Treated Neuronal Cells. J. Alzheimer’s Dis..

[17] Nagata K., Takahashi M., Matsuba Y., Okuyama-Uchimura F., Sato K., Hashimoto S., Saito T., Saido T.C. (2018). Generation of App knock-in mice reveals deletion mutations protective against Alzheimer’s disease-like pathology. Nat. Commun..

[18] McQuade A., Kang Y.J., Hasselmann J., Jairaman A., Sotelo A., Coburn M., Shabestari S.K., Chadarevian J.P., Fote G., Tu C.H. (2020). Gene expression and functional deficits underlie TREM2-knockout microglia responses in human models of Alzheimer’s disease. Nat. Commun..

[19] Barman N.C., Khan N.M., Islam M., Nain Z., Roy R.K., Haque A., Barman S.K. (2020). CRISPR-Cas9: A Promising Genome Editing Therapeutic Tool for Alzheimer’s Disease-A Narrative Review. Neurol. Ther..

[20] Komor A.C., Badran A.H., Liu D.R. (2016). CRISPR-Based Technologies for the Manipulation of Eukaryotic Genomes. Cell.

[21] Xu C.L., Ruan M.Z.C., Mahajan V.B., Tsang S.H. (2019). Viral Delivery Systems for CRISPR. Viruses.

[22] Dorszewska J., Prendecki M., Oczkowska A., Dezor M., Kozubski W. (2016). Molecular Basis of Familial and Sporadic Alzheimer’s Disease. Curr. Alzheimer Res..

[23] Escamilla-Ayala A., Wouters R., Sannerud R., Annaert W. (2020). Contribution of the Presenilins in the cell biology, structure and function of gamma-secretase. Semin. Cell Dev. Biol..

[24] Serrano-Pozo A., Das S., Hyman B.T. (2021). APOE and Alzheimer’s disease: Advances in genetics, pathophysiology, and therapeutic approaches. Lancet Neurol..

[25] Akhtar A., Singh S., Kaushik R., Awasthi R., Behl T. (2024). Types of memory, dementia, Alzheimer’s disease, and their various pathological cascades as targets for potential pharmacological drugs. Ageing Res. Rev..

[26] Ye T., Duan Y., Tsang H.W.S., Xu H., Chen Y., Cao H., Chen Y., Fu A.K.Y., Ip N.Y. (2021). Efficient manipulation of gene dosage in human iPSCs using CRISPR/Cas9 nickases. Commun. Biol..

[27] Inoue K. (2021). CRISPR-activated patient fibroblasts for modeling of familial Alzheimer’s disease. Neurosci. Res..

[28] Zhong M.Z., Peng T., Duarte M.L., Wang M., Cai D. (2024). Updates on mouse models of Alzheimer’s disease. Mol. Neurodegener..

[29] Lin Y.T., Seo J., Gao F., Feldman H.M., Wen H.L., Penney J., Cam H.P., Gjoneska E., Raja W.K., Cheng J. (2018). APOE4 Causes Widespread Molecular and Cellular Alterations Associated with Alzheimer’s Disease Phenotypes in Human iPSC-Derived Brain Cell Types. Neuron.

[30] Victor M.B., Leary N., Luna X., Meharena H.S., Scannail A.N., Bozzelli P.L., Samaan G., Murdock M.H., von Maydell D., Effenberger A.H. (2022). Lipid accumulation induced by APOE4 impairs microglial surveillance of neuronal-network activity. Cell Stem Cell.

[31] Knupp A., Mishra S., Martinez R., Braggin J.E., Szabo M., Kinoshita C., Hailey D.W., Small S.A., Jayadev S., Young J.E. (2020). Depletion of the AD Risk Gene SORL1 Selectively Impairs Neuronal Endosomal Traffic Independent of Amyloidogenic APP Processing. Cell Rep..

[32] Xiang X., Piers T.M., Wefers B., Zhu K., Mallach A., Brunner B., Kleinberger G., Song W., Colonna M., Herms J. (2018). The Trem2 R47H Alzheimer’s risk variant impairs splicing and reduces Trem2 mRNA and protein in mice but not in humans. Mol. Neurodegener..

[33] Kawatani K., Holm M.L., Starling S.C., Martens Y.A., Zhao J., Lu W., Ren Y., Li Z., Jiang P., Jiang Y. (2024). ABCA7 deficiency causes neuronal dysregulation by altering mitochondrial lipid metabolism. Mol. Psychiatry.

[34] Chadarevian J.P., Davtyan H., Chadarevian A.L., Nguyen J., Capocchi J.K., Le L., Escobar A., Chadarevian T., Mansour K., Deynega E. (2025). Harnessing human iPSC-microglia for CNS-wide delivery of disease-modifying proteins. Cell Stem Cell.

[35] Cheng-Hathaway P.J., Reed-Geaghan E.G., Jay T.R., Casali B.T., Bemiller S.M., Puntambekar S.S., von Saucken V.E., Williams R.Y., Karlo J.C., Moutinho M. (2018). The Trem2 R47H variant confers loss-of-function-like phenotypes in Alzheimer’s disease. Mol. Neurodegener..

[36] Green N.F.O., Sutton G.J., Perez-Burillo J., Wang J., Bagot S., Danon H.G., Walsh K., Gokool A., Miles S.A., Yang G. (2026). CRISPRi screening in cultured human astrocytes uncovers distal enhancers controlling genes dysregulated in Alzheimer’s disease. Nat. Neurosci..

[37] Cardona C.L., Wei L., Kim J., Angeles E., Singh G., Chen S., Patel R., Ifediora N., Canoll P., Teich A.F. (2025). High throughput identification of genetic regulators of microglial inflammatory processes in Alzheimer’s disease. J. Neuroinflamm..

[38] Meier S., Larsen A.S.G., Wanke F., Mercado N., Mei A., Takacs L., Mracsko E.S., Collin L., Kampmann M., Roudnicky F. (2025). An efficient, non-viral arrayed CRISPR screening platform for iPSC-derived myeloid and microglia models. Stem Cell Rep..

[39] Sanchez C.G., Acker C.M., Gray A., Varadarajan M., Song C., Cochran N.R., Paula S., Lindeman A., An S., McAllister G. (2021). Genome-wide CRISPR screen identifies protein pathways modulating tau protein levels in neurons. Commun. Biol..

[40] Kim M.S., Ra E.A., Kweon S.H., Seo B.A., Ko H.S., Oh Y., Lee G. (2023). Advanced human iPSC-based preclinical model for Parkinson’s disease with optogenetic alpha-synuclein aggregation. Cell Stem Cell.

[41] Meng X., Reis N., Bassik M.C., Pasca S.P. (2025). CRISPR screens in human neural organoids and assembloids. Nat. Protoc..

[42] Mrza M.A., He J., Wang Y. (2024). Integration of iPSC-Derived Microglia into Brain Organoids for Neurological Research. Int. J. Mol. Sci..

[43] Raffaele I., Cipriano G.L., Anchesi I., Oddo S., Silvestro S. (2025). CRISPR/Cas9 and iPSC-Based Therapeutic Approaches in Alzheimer’s Disease. Antioxidants.

[44] Gyorgy B., Loov C., Zaborowski M.P., Takeda S., Kleinstiver B.P., Commins C., Kastanenka K., Mu D., Volak A., Giedraitis V. (2018). CRISPR/Cas9 Mediated Disruption of the Swedish APP Allele as a Therapeutic Approach for Early-Onset Alzheimer’s Disease. Mol. Ther. Nucleic Acids.

[45] Konstantinidis E., Molisak A., Perrin F., Streubel-Gallasch L., Fayad S., Kim D.Y., Petri K., Aryee M.J., Aguilar X., Gyorgy B. (2022). CRISPR-Cas9 treatment partially restores amyloid-beta 42/40 in human fibroblasts with the Alzheimer’s disease PSEN 1 M146L mutation. Mol. Ther. Nucleic Acids.

[46] Ortiz-Virumbrales M., Moreno C.L., Kruglikov I., Marazuela P., Sproul A., Jacob S., Zimmer M., Paull D., Zhang B., Schadt E.E. (2017). CRISPR/Cas9-Correctable mutation-related molecular and physiological phenotypes in iPSC-derived Alzheimer’s PSEN2 (N141I) neurons. Acta Neuropathol. Commun..

[47] Ng B., Vowles J., Bertherat F., Abey A., Kilfeather P., Beccano-Kelly D., Stefana M.I., O’Brien D.P., Bengoa-Vergniory N., Carling P.J. (2024). Tau depletion in human neurons mitigates Abeta-driven toxicity. Mol. Psychiatry.

[48] Mesa H., Zhang E.Y., Wang Y., Zhang Q. (2024). Human neurons lacking amyloid precursor protein exhibit cholesterol-associated developmental and presynaptic deficits. J. Cell. Physiol..

[49] Guyon A., Rousseau J., Begin F.G., Bertin T., Lamothe G., Tremblay J.P. (2021). Base editing strategy for insertion of the A673T mutation in the APP gene to prevent the development of AD in vitro. Mol. Ther. Nucleic Acids.

[50] Zhao J., Fu Y., Yamazaki Y., Ren Y., Davis M.D., Liu C.C., Lu W., Wang X., Chen K., Cherukuri Y. (2020). APOE4 exacerbates synapse loss and neurodegeneration in Alzheimer’s disease patient iPSC-derived cerebral organoids. Nat. Commun..

[51] Jadhav V.S., Lin P.B.C., Pennington T., Di Prisco G.V., Jannu A.J., Xu G., Moutinho M., Zhang J., Atwood B.K., Puntambekar S.S. (2020). Trem2 Y38C mutation and loss of Trem2 impairs neuronal synapses in adult mice. Mol. Neurodegener..

[52] Zhao H., Ji Q., Wu Z., Wang S., Ren J., Yan K., Wang Z., Hu J., Chu Q., Hu H. (2022). Destabilizing heterochromatin by APOE mediates senescence. Nat. Aging.

[53] Lee S.H., Meilandt W.J., Xie L., Gandham V.D., Ngu H., Barck K.H., Rezzonico M.G., Imperio J., Lalehzadeh G., Huntley M.A. (2021). Trem2 restrains the enhancement of tau accumulation and neurodegeneration by beta-amyloid pathology. Neuron.

[54] Kang S.S., Kurti A., Baker K.E., Liu C.C., Colonna M., Ulrich J.D., Holtzman D.M., Bu G., Fryer J.D. (2018). Behavioral and transcriptomic analysis of Trem2-null mice: Not all knockout mice are created equal. Hum. Mol. Genet..

[55] Haq I., Ngo J.C., Roy N., Pan R.L., Nawsheen N., Chiu R., Zhang Y., Fujita M., Soni R.K., Wu X. (2024). An integrated toolkit for human microglia functional genomics. Stem Cell Res. Ther..

[56] Davis J.R., Banskota S., Levy J.M., Newby G.A., Wang X., Anzalone A.V., Nelson A.T., Chen P.J., Hennes A.D., An M. (2024). Efficient prime editing in mouse brain, liver and heart with dual AAVs. Nat. Biotechnol..

[57] Buchholz S., Kabbani M.A.A., Bell-Simons M., Kluge L., Cagmak C., Klimek J., Haag N., Iohan L.C., Coulon A., Costa M.R. (2025). The tau isoform 1N4R confers vulnerability of MAPT knockout human iPSC-derived neurons to amyloid beta and phosphorylated tau-induced neuronal dysfunction. Alzheimer’s Dement..

[58] Poon A., Schmid B., Pires C., Nielsen T.T., Hjermind L.E., Nielsen J.E., Holst B., Hyttel P., Freude K.K. (2016). Generation of a gene-corrected isogenic control hiPSC line derived from a familial Alzheimer’s disease patient carrying a L150P mutation in presenilin 1. Stem Cell Res..

[59] Kwart D., Gregg A., Scheckel C., Murphy E.A., Paquet D., Duffield M., Fak J., Olsen O., Darnell R.B., Tessier-Lavigne M. (2019). A Large Panel of Isogenic APP and PSEN1 Mutant Human iPSC Neurons Reveals Shared Endosomal Abnormalities Mediated by APP beta-CTFs, Not Abeta. Neuron.

[60] Oksanen M., Petersen A.J., Naumenko N., Puttonen K., Lehtonen S., Gubert Olive M., Shakirzyanova A., Leskela S., Sarajarvi T., Viitanen M. (2017). PSEN1 Mutant iPSC-Derived Model Reveals Severe Astrocyte Pathology in Alzheimer’s Disease. Stem Cell Rep..

[61] Xu L., Yao S., Ding Y.E., Xie M., Feng D., Sha P., Tan L., Bei F., Yao Y. (2024). Designing and optimizing AAV-mediated gene therapy for neurodegenerative diseases: From bench to bedside. J. Transl. Med..

[62] Devinsky O., Coller J., Ahrens-Nicklas R., Liu X.S., Ahituv N., Davidson B.L., Bishop K.M., Weiss Y., Mingorance A. (2025). Gene therapies for neurogenetic disorders. Trends Mol. Med..

[63] Zou Y., Sun X., Yang Q., Zheng M., Shimoni O., Ruan W., Wang Y., Zhang D., Yin J., Huang X. (2022). Blood-brain barrier-penetrating single CRISPR-Cas9 nanocapsules for effective and safe glioblastoma gene therapy. Sci. Adv..

[64] Moosavi S.G., Rahiman N., Jaafari M.R., Arabi L. (2025). Lipid nanoparticle (LNP) mediated mRNA delivery in neurodegenerative diseases. J. Control. Release.

[65] Foldvari M., Chen D.W., Nafissi N., Calderon D., Narsineni L., Rafiee A. (2016). Non-viral gene therapy: Gains and challenges of non-invasive administration methods. J. Control. Release.

[66] Sweeney M.D., Zhao Z., Montagne A., Nelson A.R., Zlokovic B.V. (2019). Blood-Brain Barrier: From Physiology to Disease and Back. Physiol. Rev..

[67] Crudele J.M., Chamberlain J.S. (2018). Cas9 immunity creates challenges for CRISPR gene editing therapies. Nat. Commun..

[68] van der Loo J.C., Wright J.F. (2016). Progress and challenges in viral vector manufacturing. Hum. Mol. Genet..

[69] Deverman B.E., Pravdo P.L., Simpson B.P., Kumar S.R., Chan K.Y., Banerjee A., Wu W.L., Yang B., Huber N., Pasca S.P. (2016). Cre-dependent selection yields AAV variants for widespread gene transfer to the adult brain. Nat. Biotechnol..

[70] Chan K.Y., Jang M.J., Yoo B.B., Greenbaum A., Ravi N., Wu W.L., Sanchez-Guardado L., Lois C., Mazmanian S.K., Deverman B.E. (2017). Engineered AAVs for efficient noninvasive gene delivery to the central and peripheral nervous systems. Nat. Neurosci..

[71] Pacak C.A., Mah C.S., Thattaliyath B.D., Conlon T.J., Lewis M.A., Cloutier D.E., Zolotukhin I., Tarantal A.F., Byrne B.J. (2006). Recombinant adeno-associated virus serotype 9 leads to preferential cardiac transduction in vivo. Circ. Res..

[72] Zhi S., Chen Y., Wu G., Wen J., Wu J., Liu Q., Li Y., Kang R., Hu S., Wang J. (2022). Dual-AAV delivering split prime editor system for in vivo genome editing. Mol. Ther..

[73] Doman J.L., Pandey S., Neugebauer M.E., An M., Davis J.R., Randolph P.B., McElroy A., Gao X.D., Raguram A., Richter M.F. (2023). Phage-assisted evolution and protein engineering yield compact, efficient prime editors. Cell.

[74] Tang Y., Han T., Everts M., Zhu Z.B., Gillespie G.Y., Curiel D.T., Wu H. (2007). Directing adenovirus across the blood-brain barrier via melanotransferrin (P97) transcytosis pathway in an in vitro model. Gene Ther..

[75] Humbel M., Ramosaj M., Zimmer V., Regio S., Aeby L., Moser S., Boizot A., Sipion M., Rey M., Deglon N. (2021). Maximizing lentiviral vector gene transfer in the CNS. Gene Ther..

[76] Raguram A., Banskota S., Liu D.R. (2022). Therapeutic in vivo delivery of gene editing agents. Cell.

[77] Kim S., Kim D., Cho S.W., Kim J., Kim J.S. (2014). Highly efficient RNA-guided genome editing in human cells via delivery of purified Cas9 ribonucleoproteins. Genome Res..

[78] Yin H., Song C.Q., Dorkin J.R., Zhu L.J., Li Y., Wu Q., Park A., Yang J., Suresh S., Bizhanova A. (2016). Therapeutic genome editing by combined viral and non-viral delivery of CRISPR system components in vivo. Nat. Biotechnol..

[79] Liu J., Chang J., Jiang Y., Meng X., Sun T., Mao L., Xu Q., Wang M. (2019). Fast and Efficient CRISPR/Cas9 Genome Editing In Vivo Enabled by Bioreducible Lipid and Messenger RNA Nanoparticles. Adv. Mater..

[80] Wang H.X., Li M., Lee C.M., Chakraborty S., Kim H.W., Bao G., Leong K.W. (2017). CRISPR/Cas9-Based Genome Editing for Disease Modeling and Therapy: Challenges and Opportunities for Nonviral Delivery. Chem. Rev..

[81] Lu C.T., Zhao Y.Z., Wong H.L., Cai J., Peng L., Tian X.Q. (2014). Current approaches to enhance CNS delivery of drugs across the brain barriers. Int. J. Nanomed..

[82] Oller-Salvia B., Sanchez-Navarro M., Giralt E., Teixido M. (2016). Blood-brain barrier shuttle peptides: An emerging paradigm for brain delivery. Chem. Soc. Rev..

[83] Zhang X., Chai Z., Lee Dobbins A., Itano M.S., Askew C., Miao Z., Niu H., Samulski R.J., Li C. (2022). Customized blood-brain barrier shuttle peptide to increase AAV9 vector crossing the BBB and augment transduction in the brain. Biomaterials.

[84] Zhang X., He T., Chai Z., Samulski R.J., Li C. (2018). Blood-brain barrier shuttle peptides enhance AAV transduction in the brain after systemic administration. Biomaterials.

[85] Hsu P.H., Wei K.C., Huang C.Y., Wen C.J., Yen T.C., Liu C.L., Lin Y.T., Chen J.C., Shen C.R., Liu H.L. (2013). Noninvasive and targeted gene delivery into the brain using microbubble-facilitated focused ultrasound. PLoS ONE.

[86] Hynynen K., McDannold N., Sheikov N.A., Jolesz F.A., Vykhodtseva N. (2005). Local and reversible blood-brain barrier disruption by noninvasive focused ultrasound at frequencies suitable for trans-skull sonications. Neuroimage.

[87] Stavarache M.A., Petersen N., Jurgens E.M., Milstein E.R., Rosenfeld Z.B., Ballon D.J., Kaplitt M.G. (2019). Safe and stable noninvasive focal gene delivery to the mammalian brain following focused ultrasound. J. Neurosurg..

[88] Alonso A., Reinz E., Leuchs B., Kleinschmidt J., Fatar M., Geers B., Lentacker I., Hennerici M.G., de Smedt S.C., Meairs S. (2014). Focal delivery of AAV2/1-transgenes into the rat brain by localized ultrasound-induced BBB Opening. Ann. Neurosci..

[89] Soni N., Kar I., Narendrasinh J.D., Shah S.K., Konathala L., Mohamed N., Kachhadia M.P., Chaudhary M.H., Dave V.A., Kumar L. (2024). Role and application of CRISPR-Cas9 in the management of Alzheimer’s disease. Ann. Med. Surg..

[90] Musunuru K., Chadwick A.C., Mizoguchi T., Garcia S.P., DeNizio J.E., Reiss C.W., Wang K., Iyer S., Dutta C., Clendaniel V. (2021). In vivo CRISPR base editing of PCSK9 durably lowers cholesterol in primates. Nature.

[91] Gillmore J.D., Gane E., Taubel J., Kao J., Fontana M., Maitland M.L., Seitzer J., O’Connell D., Walsh K.R., Wood K. (2021). CRISPR-Cas9 In Vivo Gene Editing for Transthyretin Amyloidosis. N. Engl. J. Med..

